# Increased frequency of CD4^+^CD57^+^ senescent T cells in patients with newly diagnosed acute heart failure: exploring new pathogenic mechanisms with clinical relevance

**DOI:** 10.1038/s41598-019-49332-5

**Published:** 2019-09-09

**Authors:** Jong-Chan Youn, Min Kyung Jung, Hee Tae Yu, Ji-Soo Kwon, Jeong-Eun Kwak, Su-Hyung Park, In-Cheol Kim, Myung-Soo Park, Sun Ki Lee, Suk-Won Choi, Seongwoo Han, Kyu-Hyung Ryu, Seok-Min Kang, Eui-Cheol Shin

**Affiliations:** 1 Division of Cardiology, Department of Internal Medicine, Seoul St. Mary’s Hospital, College of Medicine, The Catholic University of Korea, Seoul, Republic of Korea; 2 Laboratory of Immunology and Infectious Diseases, Graduate School of Medical Science and Engineering, Korea Advanced Institute of Science and Technology (KAIST), Daejeon, Republic of Korea; 3 Division of Cardiology, Severance Cardiovascular Hospital, Yonsei University College of Medicine, Seoul, Republic of Korea; 4 BioMedical Science and Engineering Interdisciplinary Program, Korea Advanced Institute of Science and Technology (KAIST), Daejeon, Republic of Korea; 5 Laboratory of Translational Immunology and Vaccinology, Graduate School of Medical Science and Engineering, Korea Advanced Institute of Science and Technology (KAIST), Daejeon, Republic of Korea; 6 Division of Cardiology, Keimyung University Dongsan Medical Center, Daegu, Republic of Korea; 7 Division of Cardiology, Dongtan Sacred Heart Hospital, Hallym University College of Medicine, Hwaseong, Republic of Korea

**Keywords:** Translational immunology, Heart failure

## Abstract

Recent animal studies showed T cells have a direct pathogenic role in the development of heart failure (HF). However, which subsets of T cells contribute to human HF pathogenesis and progression remains unclear. We characterized immunologic properties of various subsets of T cells and their clinical implications in human HF. Thirty-eight consecutive patients with newly diagnosed acute HF (21 males, mean age 66 ± 16 years) and 38 healthy control subjects (21 males, mean age 62 ± 12 years) were enrolled. We found that pro-inflammatory mediators, including CRP, IL-6 and IP-10 and the frequencies of CD57^+^ T cells in the CD4^+^ T cell population were significantly elevated in patients with acute HF compared to control subjects. A functional analysis of T cells from patients with acute HF revealed that the CD4^+^CD57^+^ T cell population exhibited a higher frequency of IFN-γ- and TNF-α- producing cells compared to the CD4^+^CD57^−^ T cell population. Furthermore, the frequency of CD4^+^CD57^+^ T cells at baseline and its elevation at the six-month follow-up were significantly related with the development of cardiovascular (CV) events, which were defined as CV mortality, cardiac transplantation, or rehospitalization due to HF exacerbation. In conclusion, CD4^+^CD57^+^ senescent T cells showed more inflammatory features and polyfunctionality and were associated with clinical outcome in patients with acute HF. More detailed study for senescent T cells might offer new opportunities for the prevention and treatment of human HF.

## Introduction

Heart failure (HF) is an important cardiovascular syndrome with significant morbidity and high mortality rates. The increasing prevalence of HF has contributed to rapidly expanding health care costs^1,2^. Since the early 1990’s, inflammation and immunological responses have been recognized as clinically relevant features of HF^3^. Numerous studies have reported that circulating levels of tumour necrosis factor-α (TNF-α) were correlated with HF severity and prognosis. Based on those observations, large-scale clinical trials were launched that targeted TNF-α with infliximab or etanercept^4,5^. Those trials were deemed unsuccessful, and they were terminated prematurely, due to the lack of clinical benefit, and in some patients, disease progression^4,5^. Although TNF-α blockers failed in trials, studies continue to investigate more sophisticated and specific immunological aspects of HF pathogenesis^2^.

Recently, the pathologic role of T cells in the development of HF was demonstrated in two mouse models; one was a non-ischaemic, pressure-overload HF model^6,7^ and the other was an ischaemic HF model^8^. These studies showed that T cells have a direct pathogenic role in the development of HF, not just as an epiphenomenon. However, which subsets of T cells contribute to HF pathogenesis and progression remains unclear. Therefore, we designed our clinical study to evaluate which subset of T cells has pathological immune characteristics and what their clinical implication is in patients with acute HF.

Memory T cells undergo replicative senescence via repetitive proliferation by antigen stimulation. Senescent T cells have the ability to produce large quantities of pro-inflammatory cytokines and cytotoxic mediators and have been implicated in the pathogenesis of many chronic inflammatory diseases, including cardiovascular diseases^9–11^. In humans, senescent T cells typically lose expression of CD28 and acquire the expression of CD57 with repetitive replication. Therefore the frequency of CD28^null^ and CD57^+^ T cells in the peripheral blood increases with age^9–11^. A growing body of evidence suggests that senescent T cells also have pathogenic potential in cardiovascular diseases, such as hypertension, atherosclerosis, and myocardial infarction, underscoring the detrimental roles of these cells in various chronic inflammatory responses. We previously found that CD8^+^CD57^+^ T cells were associated with the development of hypertension and the progression of myocardial infarction^12–14^. Though which subsets of T cells contribute to deleterious inflammation in HF progression is unclear, we hypothesized that senescent T cells may be involved in the progression of HF, as hypertension, myocardial infarction, and HF are all tied to the aging process.

In the present study, we focused on CD28^null^ and CD57^+^ replicative senescent T cells isolated from patients with acute HF. We examined immunological characteristics and clinical implication of various subsets of T cells, particularly CD4^+^CD57^+^ senescent T cells. We found that patients with acute HF had elevated frequencies of CD4^+^CD57^+^ senescent T cells, and this increase was associated with clinical outcome.

## Materials and Methods

### Study population

The present study prospectively enrolled 38 newly diagnosed consecutive patients with acute HF (12 ischemic, 26 non-ischemic) at the Severance Cardiovascular Hospital, from October 2014 to August 2015. Patients were eligible for the study when they displayed rapid onset of signs or symptoms of HF and one of the following criteria: (i) lung congestion or (ii) objective findings of left ventricular systolic dysfunction or structural heart disease. Lung congestion was defined as ‘congestion’ on a chest X-ray or rales in a physical examination. Patients that had recovered from acute HF and were ambulatory during hospitalization were eligible for the study. Patients were excluded from the study when they had a history of overt chronic inflammatory diseases, such as rheumatoid arthritis or autoimmune disease, and/or were taking anti-inflammatory medications. The primary endpoint included a cardiovascular (CV) event, defined as the composite of death, urgent cardiac transplantation, or rehospitalization due to worsening heart failure. In the Yonsei Cardiovascular Genome Centre, we enrolled 38 age- and sex-matched healthy control subjects that had no history of HF. These controls were evaluated with routine physical examinations, laboratory tests, and radiological examinations. The baseline characteristics of the acute HF and control groups are summarized in Table 1. This study was carried out in accordance with the ethical guidelines of the Declaration of Helsinki and was approved by the institutional review board (IRB) of Yonsei University College of Medicine, Severance Hospital, Seoul, Republic of Korea (IRB number 4-2012-0027), and written informed consent was obtained from all of the participants^15^.

**Table 1:** Clinical characteristics, laboratory findings, and senescent T cell subsets for patients with newly diagnosed acute HF and age- and sex-matched control subjects.

	Acute HF (n = 38)	Control (n = 38)	p-Value
Age (y)	66 ± 16	62 ± 12	0.208
Male: Female (N, %)	21:17 (55.3%:44.7%)	21:17 (55.3%:44.7%)	—
BMI (kg/m^2^)	23.8 ± 4.2	24.8 ± 4.3	0.082
WBC (×10^3^/μl)	8.71 ± 3.86	5.94 ± 1.58	<0.001^*^
Haemoglobin (g/dl)	13.7 ± 2.3	13.9 ± 1.3	0.604
BUN (mg/dl)	23.9 ± 12.5	18.2 ± 5.1	0.014^*^
Creatinine (mg/dl)	1.16 ± 0.55	1.03 ± 0.17	0.176
Total cholesterol (mg/dl)	154.9 ± 59.1	178.2 ± 46.1	0.069
Albumin (mg/dl)	3.7 ± 0.4	4.5 ± 0.3	<0.001^*^
CRP (mg/l)	6.11 ± 6.80	1.97 ± 3.14	0.001^*^
IL-6 (pg/ml)	7.30 ± 10.17	1.43 ± 1.98	0.001^*^
TNF-α (pg/ml)	7.39 ± 10.17	3.45 ± 6.30	0.272
MIG (pg/ml)	362.8 ± 774.2	115.6 ± 221.0	0.066
IP-10 (pg/ml)	248.1 ± 169.2	101.2 ± 58.6	<0.001^*^
MIP-1α (pg/ml)	28.9 ± 58.7	23.5 ± 25.7	0.610
CD4^+^CD28^null^ T cell fraction in CD4^+^ T cell population (%)	2.8 ± 3.6	1.4 ± 1.2	0.035^*^
CD4^+^CD57^+^ T cell fraction in CD4^+^ T cell population (%)	5.0 ± 3.9	2.5 ± 1.5	0.001^*^
CD8^+^CD28^null^ T cell fraction in CD8^+^ T cell population (%)	38.3 ± 17.5	32.7 ± 12.1	0.118
CD8^+^CD57^+^ T cell fraction in CD8^+^ T cell population (%)	34.8 ± 14.7	31.8 ± 12.0	0.338

Values represent the number (%) of patients or the mean ± standard deviation, as indicated. ^*^p-values < 0.05 were considered significant. HF, heart failure; BMI, body mass index; WBC, white blood cell count; BUN, blood urea nitrogen; CRP, C-reactive protein; IL-6, interleukin-6; TNF-α, tumour necrosis factor-α; MIG, monokine-induced by gamma interferon; IP-10, interferon gamma-induced protein 10; MIP-1α, macrophage inflammatory protein-1α.

### Measurement of pro-inflammatory mediators

We sampled peripheral blood after patients achieved clinical stabilization, according to current HF guidelines^15,16^. Clinical stabilization was defined as readiness for discharge from the hospital: congestion was absent, and a stable, oral diuretic regimen was established for at least 48 h^15^. We collected blood samples at a relatively consistent time point, that is, on the day of or before discharge^15^. The concentration of C-reactive protein (CRP) was measured with an enzyme-linked immunosorbent assay (R&D system, Minneapolis, MN). We performed flow cytometry with the BD cytometric bead array kit (BD Biosciences, San Jose, CA) to measure other pro-inflammatory mediators, including interleukin-6 (IL-6), TNF-α, monokine-induced by gamma interferon (MIG), interferon gamma-induced protein 10 (IP-10), and macrophage inflammatory protein 1α (MIP1α). Samples were processed according to the manufacturer’s instructions. Mixed capture beads (50 μl) and each serum sample (50 μl) were incubated for 1 h at room temperature (RT), and then mixed phycoerythrin detection reagents (50 μl) were added to the bead-sample mixture and incubated for 2 h at RT^15^. The samples were washed and assessed with an LSR II Flow Cytometer (BD Biosciences) and then the data were analysed with FlowJo software, version 9.2 for Mac (TreeStar, Ashland, OR)^15^.

### Immunophenotyping analysis of peripheral blood mononuclear cells

Peripheral blood mononuclear cells (PBMCs) were separated from whole blood using Ficoll-Hypaque (GE Healthcare, Uppsala, Sweden), and then, cryopreserved until use^15^. Cryopreserved PBMCs were thawed and analysed with flow cytometry. Briefly, cells were stained with fluorochrome-conjugated monoclonal antibodies against surface antigens for 20 min at 4 °C. The antibodies were anti-CD3-Horizon V500, anti-CD4-PE-Cy7, anti-CD8-APC-H7, anti-CD28-APC (all from BD Biosciences) and anti-CD57-Pacific blue (Biolegend, San Diego, CA). Multicolour flow cytometry was performed with an LSR II Flow Cytometer, and data was analysed with FlowJo software.

### *In vitro* stimulation of T cells and intracellular cytokine staining

PBMCs were stimulated with anti-CD3 antibody (100 ng/ml) for 6 h. Then, after 1 h of incubation, brefeldin A (GolgiPlug, BD Biosciences) and monensin (GolgiStop, BD Biosciences) were added to the culture to cause intracellular cytokine accumulation. Cells were surface-stained with anti-CD3-Horizon V500, anti-CD4-PE-Cy7, anti-CD8-APC-H7, anti-CD28-APC, and anti-CD57-Pacific blue. Then, cells were fixed and permeabilized with the Fixation/Permeabilization Buffer Kit (BD Biosciences). Cells were then stained to detect intracellular cytokines with anti-TNF-PE-Cy7 and anti-IFN-γ-FITC (all from BD Biosciences). FACS analysis was performed with an LSR II Flow Cytometer, and data were analysed with FlowJo software.

### Statistical analysis

Continuous variables are reported as the mean ± SD. Categorical variables are expressed as percentages of the group totals. Continuous variables were compared with independent t-tests, and discrete variables were compared with the chi-squared method. Intra-group comparisons were performed with the paired t-test, and the Wilcoxon signed-rank test was used to verify the results. The Kaplan-Meier method was used to assess the cumulative incidence of CV events. For cumulative CV events, the baseline frequency of CD4^+^CD57^+^ T cells was defined with a cut-off point of 3.65, according to Youden index (sensitivity 66.7% and specificity 68.4%). The statistical significance of the curves was calculated with the log-rank test. Statistical analyses were performed with SPSS 13.0. (SPSS Inc., Chicago, IL).

## Results

### Clinical, laboratory, and senescent T-cell characteristics of patients with acute HF

We compared the clinical characteristics, laboratory findings, and senescent T-cell frequencies between patients with newly diagnosed acute HF and age-and sex-matched control subjects (Table 1). Acute HF patients showed significantly increased white blood cell count (×10^3^/μl) (8.71 ± 3.86 vs. 5.94 ± 1.58, *p* < 0.001), blood urea nitrogen (mg/dl) (23.9 ± 12.5 vs. 18.2 ± 5.1, *p = *0.014) and significantly decreased albumin (mg/dl) (3.7 ± 0.4 vs. 4.5 ± 0.3, *p* < 0.001). However, there was no significant difference in body mass index, haemoglobin and total cholesterol level.

### Pro-inflammatory mediators and CD4^+^ senescent T-cell fractions are increased in patients with newly diagnosed acute HF

**Figure 1:**
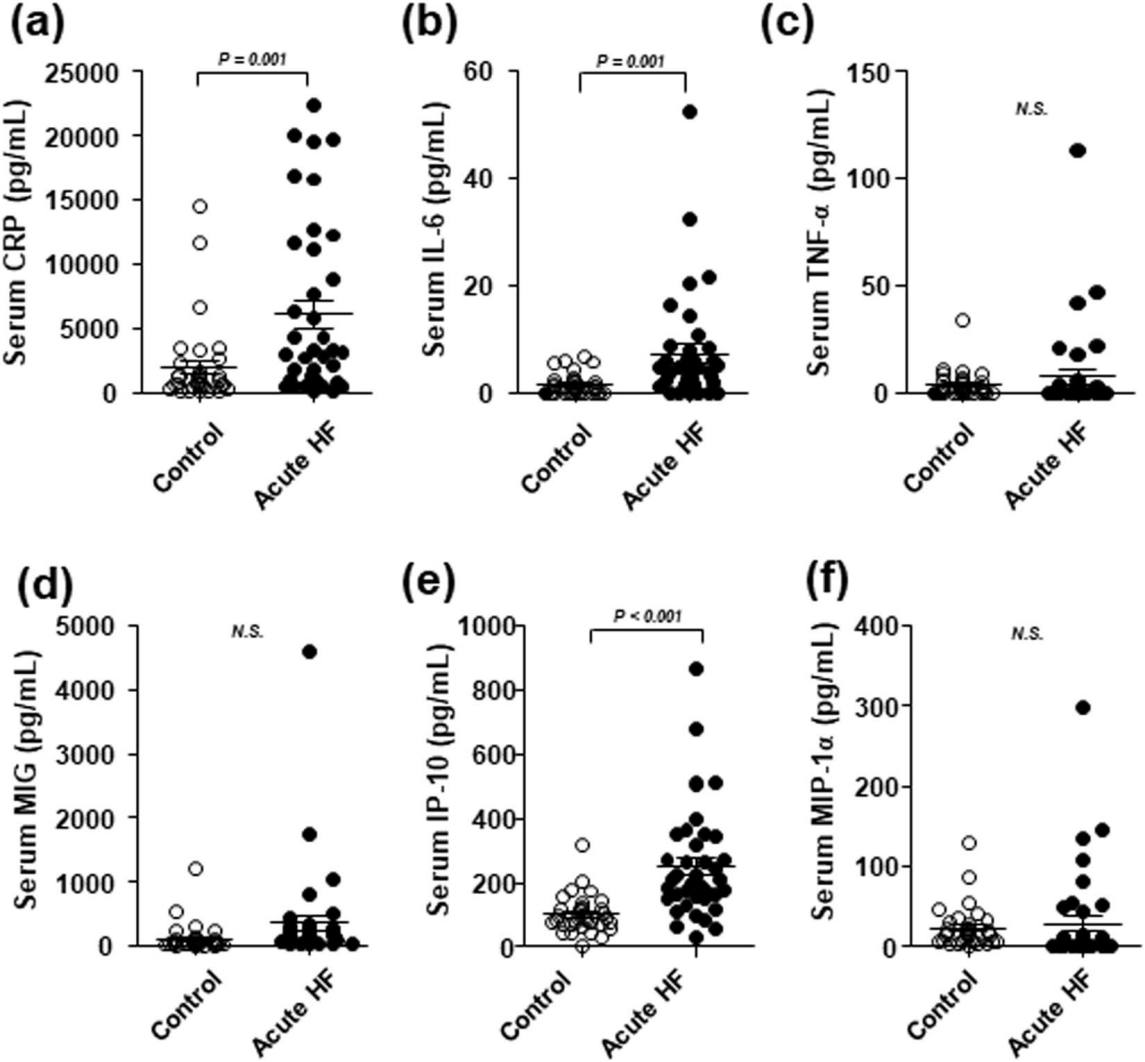
Pro-inflammatory mediators are elevated in patients with acute HF. Serum samples were obtained from healthy controls and patients with acute HF. (**a**) Enzyme-linked immunosorbent assay results show CRP concentrations. (**b**–**f**) Cytometric bead array results show (**b**) IL-6, (**c**) TNF-α, (**d**) MIG, (**e**) IP-10, and (**f**) MIP-1α concentrations. Medians and standard deviations are presented for each group. N.S., non-significant.

First, we compared serum levels of pro-inflammatory mediators between 38 patients with acute HF and 38 age- and sex-matched control subjects. Patients with acute HF showed significantly elevated levels of CRP, IL-6 and IP-10 compared to healthy controls. Serum levels of TNF-α, MIG and MIP-1α were not significantly different between groups (Fig. 1).

**Figure 2:**
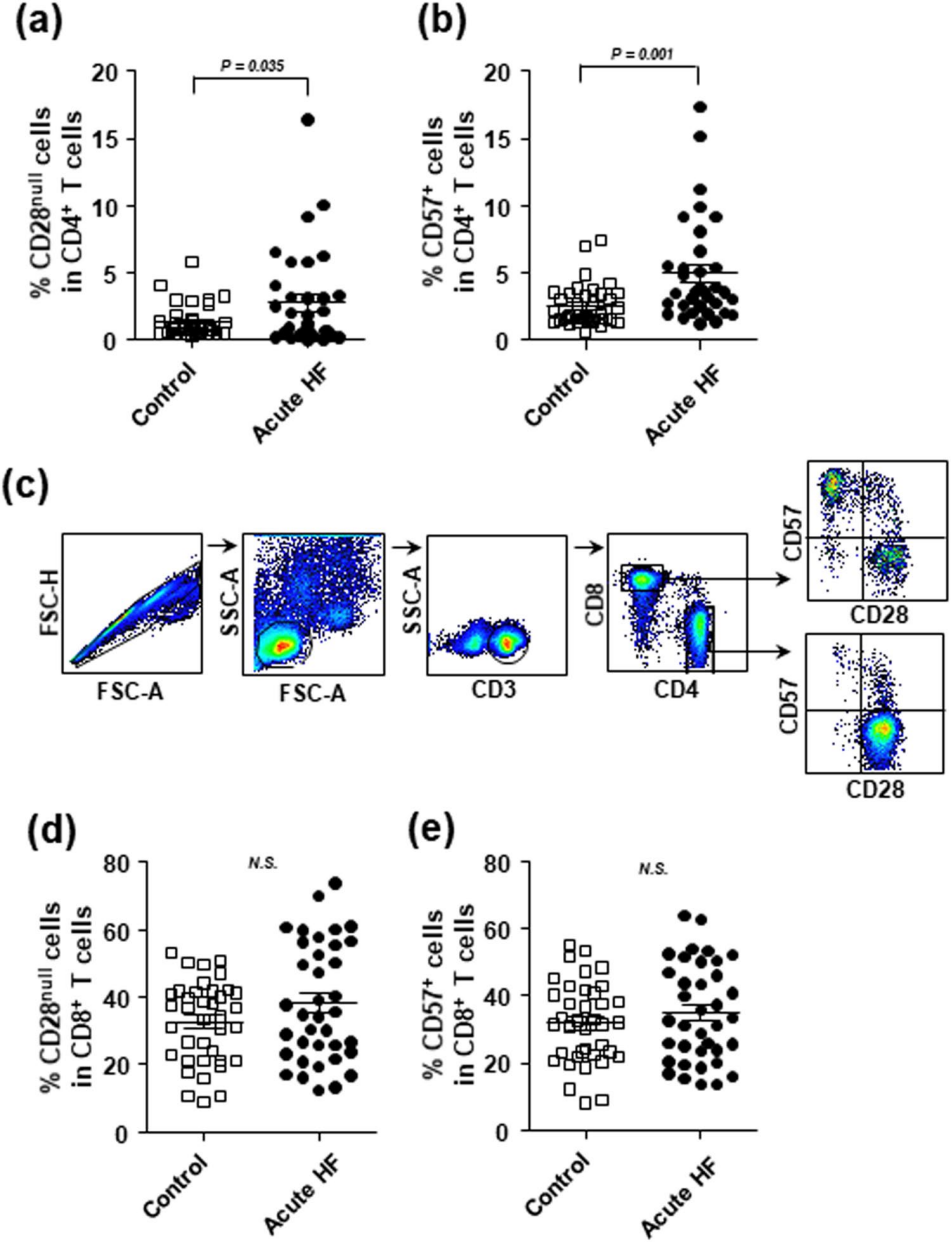
The frequencies of CD4^+^CD28^null^ and CD4^+^CD57^+^ T cells are elevated in patients with acute HF. (**a**,**b**) The percentages of (**a**) CD4^+^CD28^null^ T cells, (**b**) CD4^+^CD57^+^ T cells in the CD4^+^ T cell populations were determined in PBMCs from healthy controls and patients with acute HF. (**c**) A gating strategy for flow cytometry analysis is presented. (**d**,**e**) The percentages of (**d**) CD8^+^CD28^null^ T cells, and (**e**) CD8^+^CD57^+^ T cells in the CD8^+^ T cell populations were determined in PBMCs from healthy controls and patients with acute HF. Medians and standard deviations are presented for each group. N.S., non-significant.

Next, we compared the frequencies of CD28^null^ and CD57^+^ senescent T cells in the PBMC populations between patients with acute HF and control subjects. We found that the frequencies of CD28^null^ and CD57^+^ T cells in the CD4^+^ T cell population were significantly elevated in patients with acute HF compared to control subjects (CD4^+^CD28^null^ T cell fraction: 2.8 ± 3.6% vs. 1.4 ± 1.2%, *p* = 0.035; CD4^+^CD57^+^ T cell fraction: 5.0 ± 3.9% vs. 2.5 ± 1.5%, *p* = 0.001; Fig. 2a,b). In fact, the CD28^null^ and CD57^+^ T cell subpopulations showed considerable overlap (Fig. 2c). Conversely, the frequencies of CD28^null^ and CD57^+^ T cells in the CD8^+^ T cell population were not significantly different between the HF and control groups (CD8^+^CD28^null^ T cell fraction: 38.3 ± 17.5% vs. 32.7 ± 12.1%, *p* = 0.118; CD8^+^CD57^+^ T cell fraction: 34.8 ± 14.7% vs. 31.8 ± 12.0%, *p* = 0.338; Fig. 2d,e).

**Figure 3:**
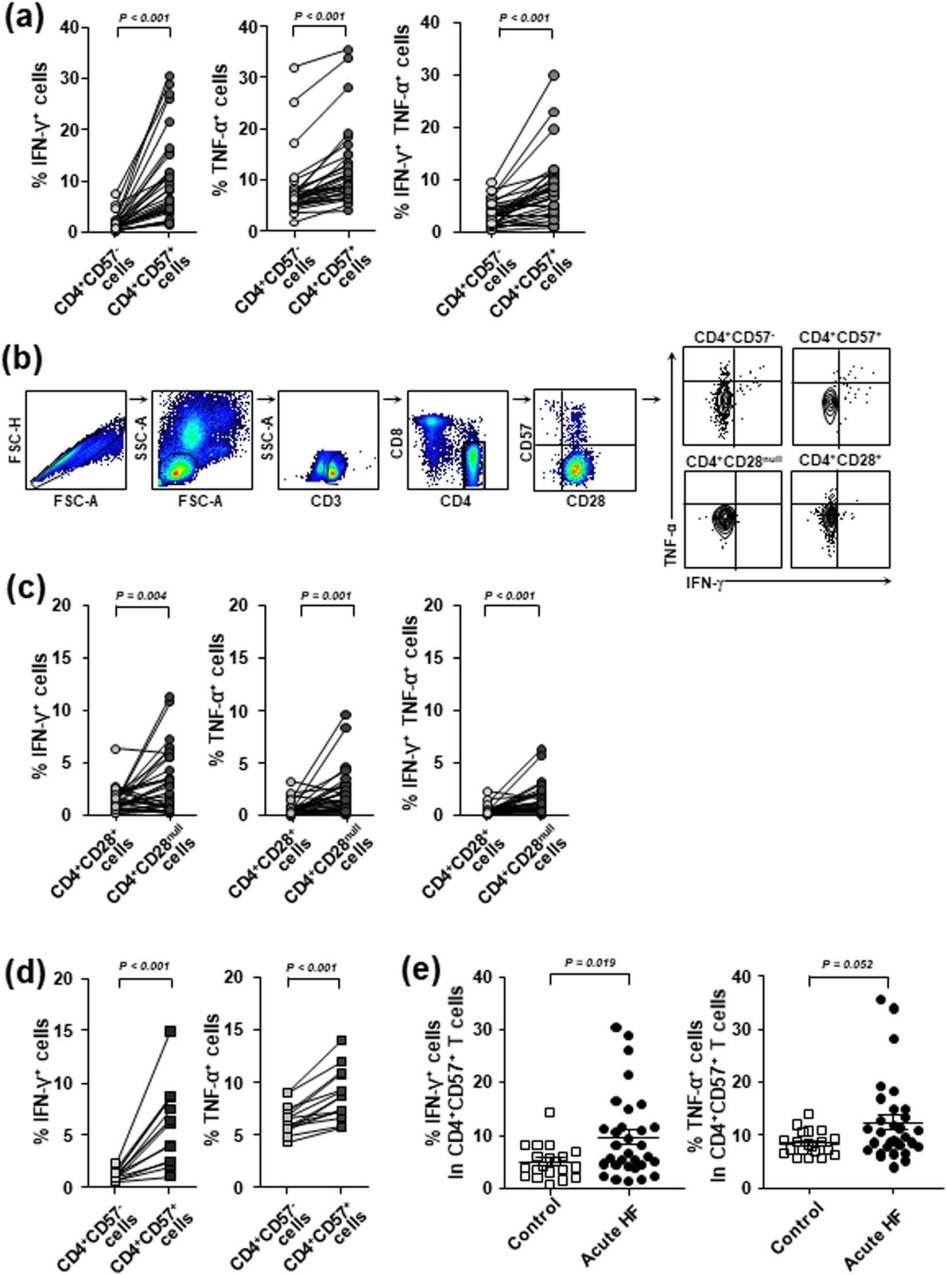
The CD4^+^CD57^+^ T cell population exhibits higher frequencies of IFN-γ- and TNF-α-producing cells than the CD4^+^CD57^−^ T cell population. PBMCs were stimulated with anti-CD3 antibody, and intracellular cytokine staining was performed to detect IFN-γ and TNF-α. (**a**) The frequencies of IFN-γ^+^, TNF-α^+^, and double positive (IFN-γ^+^TNF-α^+^) cells are shown for CD4^+^CD57^+^ T-cell and CD4^+^CD57^−^ T-cell populations from patients with acute HF. (**b**) A gating strategy for flow cytometry analysis is presented. (**c**) The frequencies of IFN-γ^+^, TNF-α^+^, and double positive (IFN-γ^+^TNF-α^+^) cells are shown for CD4^+^CD28^+^ T-cell and CD4^+^CD28^null^ T-cell populations from patients with acute HF. (**d**) The frequencies of IFN-γ^+^ and TNF-α^+^ cells are shown for CD4^+^CD57^+^ T-cell and CD4^+^CD57^−^ T-cell populations from healthy individuals. (**e**) The frequencies of IFN-γ^+^ and TNF-α ^+^ cells among the CD4^+^CD57^+^ T-cell population were compared between healthy individuals and patients with acute HF.

We analysed the functional characteristics of CD4^+^CD57^+^ T cells compared to the characteristics of CD4^+^CD57^−^ T cells from patients with acute HF. We treated cells with an anti-CD3 antibody to mimic T-cell receptor (TCR) stimulation. Subsequent intracellular cytokine staining for IFN-γ and TNF-α showed that the frequencies of IFN-γ- and TNF-α- producing cells in the CD4^+^CD57^+^ T cell population were significantly higher than those in the CD4^+^CD57^−^ T cell population (*p* < 0.001, Fig. 3a). In addition, the frequency of T cells that produced both IFN-γ and TNF-α was also significantly higher in the CD4^+^CD57^+^ T cell population compared to the CD4^+^CD57^−^ T cell population (*p* < 0.001, Fig. 3a). A gating strategy for flow cytometry analysis is presented in Fig. 3b. Similar results were obtained with CD4^+^CD28^null^ T cells. The CD4^+^CD28^null^ T cell population had higher frequencies of IFN-γ^+^ and TNFα^+^ cells than the CD4^+^CD28^+^ T cell population (*p* < 0.01, Fig. 3c). We also compared the frequencies of IFN-γ^+^ and TNF-α^+^ cells among CD4^+^CD57^+^ and CD4^+^CD57^−^ T cells from healthy individuals and found that the CD4^+^CD57^+^ T cell population has higher frequencies of IFN-γ^+^ and TNF-α^+^ cells than the CD4^+^CD57^−^ T cell population, even in healthy individuals (*p* < 0.001, Fig. 3d). However, the frequency of IFN-γ^+^ cells among CD4^+^CD57^+^ T cells was significantly higher in patients with acute HF than in healthy individuals (*p* < 0.05, Fig. 3e). A similar result was found for the frequency of TNF-α^+^ cells among CD4^+^CD57^+^ T cells although it did not reach statistical significance (*p* = 0.052, Fig. 3e).

### The frequencies of CD4^+^CD57^+^ T cells at baseline and at the 6-month follow-up were related to clinical outcome in patients with acute HF

**Figure 4:**
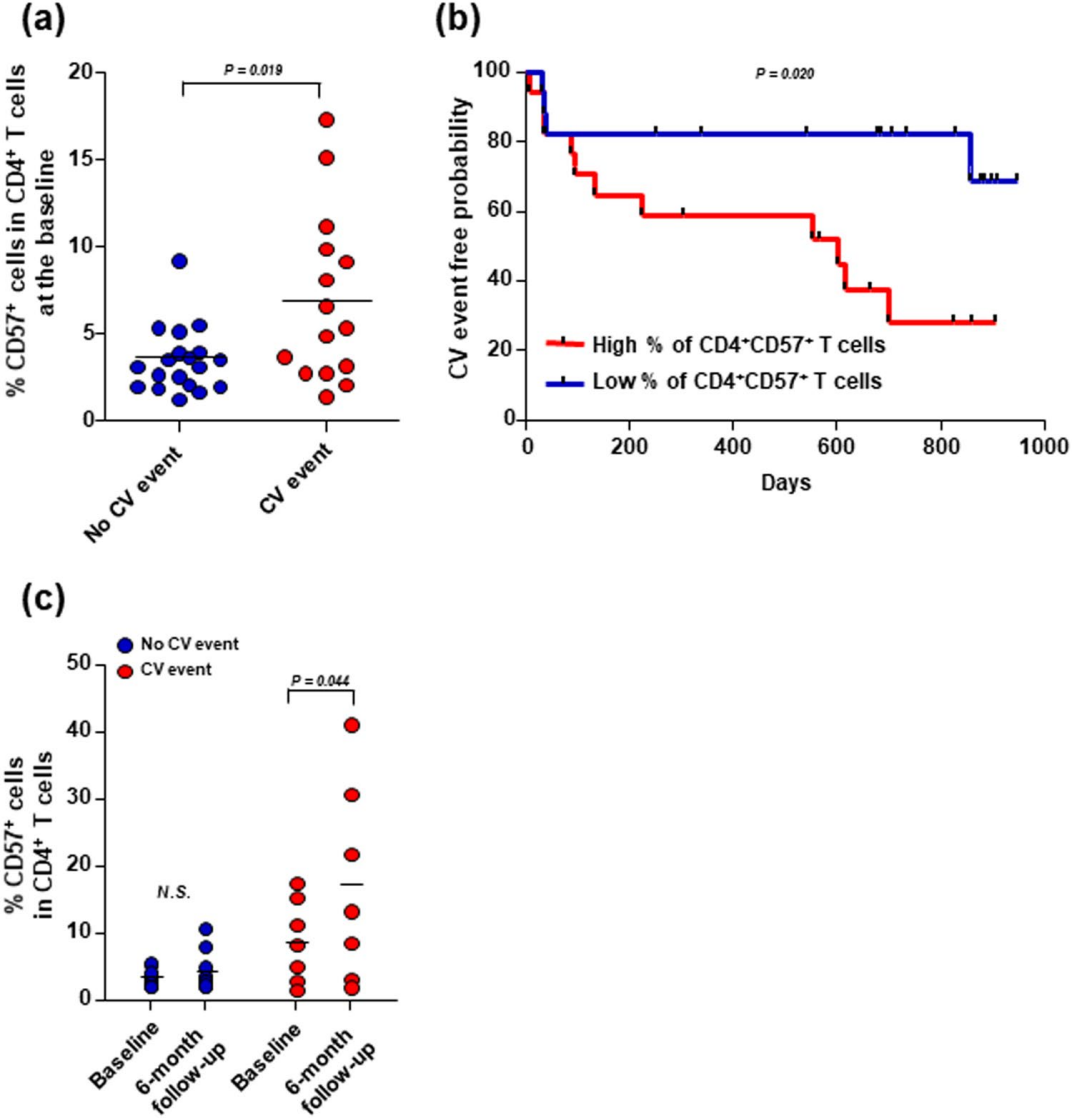
The frequencies of CD4^+^CD57^+^ T cells at baseline and at the 6-month follow-up are related to clinical outcome in patients with acute HF. (**a**) Baseline frequencies of CD4^+^CD57^+^ T cells in PBMCs from patients with acute HF were compared between those without and those with CV events. Medians are presented for each group. (**b**) Cumulative Kaplan-Meier estimates of CV events in patients with acute HF are compared between patients with different baseline CD4^+^CD57^+^ T cell frequencies (according to the Youden index). (**c**) Baseline and 6-month CD4^+^CD57^+^ T cell frequencies in PBMCs from patients with acute HF are compared between those without and those with CV events. N.S., non-significant.

We evaluated whether the elevated frequency of CD4^+^CD57^+^ T cells observed at baseline was associated with the clinical outcome in patients with acute HF. During a median follow-up period of 640 (range: 6–947) days, we observed 2 (5.3%) cardiovascular-related deaths, 2 (5.3%) urgent heart transplantations, and 12 (31.6%) rehospitalizations due to HF exacerbation. The clinical, echocardiographic, laboratory, and senescent T-cell characteristics of the 38 patients with acute HF were compared between patients that did and those that did not experience a CV event (Table 2). Patients that experienced CV events were more likely to have diabetes and showed significantly reduced renal function compared to patients with no CV events. Although these groups exhibited similar pro-inflammatory mediator levels, patients with CV events showed significantly higher frequencies of CD4^+^CD28^null^ and CD4^+^CD57^+^ T cells at baseline than patients without CV events (CD4^+^CD28^null^ T cells: 4.5 ± 4.6% vs. 1.5 ± 1.7, *p* = 0.027; CD4^+^CD57^+^ T cells: 6.9 ± 4.9% vs. 3.5 ± 1.9%, *p* = 0.019; Fig. 4a). We then divided acute HF patients into two groups according to the baseline CD4^+^CD57^+^ T cell frequency, based on the Youden index. The cumulative Kaplan-Meier estimates of CV events revealed that patients with high baseline CD4^+^CD57^+^ T cell fractions showed significantly worse clinical outcomes than patients with low baseline CD4^+^CD57^+^ T cell fractions (*p*=0.020; Fig. 4b).

**Table 2:** Clinical, echocardiographic, laboratory, and senescent T-cell characteristics in patients with acute HF that experienced or did not experience a CV event.

	CV event (N = 16, 42.1%)	No CV event (N = 22, 57.9%)	*P*-value
Age (y)	67 ± 16	65 ± 16	0.658
Male: Female (N, %)	10:6 (62.5%:37.5%)	12:10 (54.5%:45.5%)	0.439
Ischaemic aetiology (N, %)	6 (37.5%)	6 (27.3%)	0.374
Hypertension (N, %)	10 (62.5%)	15 (68.2%)	0.490
Diabetes (N, %)	9 (56.3%)	5 (22.7%)	0.047^*^
Weight (kg)	64.6 ± 10.2	61.5 ± 15.0	0.476
BMI (kg/m^2^)	22.7 ± 2.9	24.7 ± 3.8	0.452
**Echocardiographic parameters**			
LVEF (%)	26.9 ± 13.3	28.1 ± 10.2	0.742
LVEDD (mm)	65.0 ± 9.4	61.4 ± 10.7	0.310
LVESD (mm)	56.6 ± 11.5	52.8 ± 11.1	0.334
LAVI (ml/m^2^)	63.1 ± 27.2	57.6 ± 19.7	0.490
E/E’	27.8 ± 14.2	19.8 ± 10.0	0.076
**Laboratory parameters**			
WBC (×10^3^/μl)	8.98 ± 2.55	8.52 ± 4.64	0.599
Haemoglobin (g/dl)	12.8 ± 2.5	14.3 ± 1.9	0.060
Sodium (mmol/l)	137.2 ± 3.9	140.5 ± 8.2	0.143
Total cholesterol (mg/dl)	164.5 ± 83.2	147.9 ± 32.9	0.399
Albumin (g/dl)	3.7 ± 0.4	3.8 ± 0.4	0.756
eGFR (ml/min/1.73 m^2^)	58.7 ± 24.4	73.4 ± 16.4	0.046^*^
NT-proBNP (pg/ml)	8044 ± 10256	8595 ± 10036	0.869
**Pro-inflammatory mediators**			
CRP (mg/l)	8.27 ± 7.30	4.54 ± 6.11	0.096
IL-6 (pg/ml)	8.63 ± 8.57	6.33 ± 11.29	0.499
TNF-α (pg/ml)	4.98 ± 12.3	9.14 ± 25.4	0.549
MIG (pg/ml)	564.5 ± 1108.7	216.1 ± 352.9	0.241
IP-10 (pg/ml)	228.3 ± 142.0	262.4 ± 188.4	0.547
MIP-1α (pg/ml)	27.8 ± 41.6	29.7 ± 69.5	0.923
**Medications at discharge**			
RAS blockers (N, %)	12 (75.0%)	17 (77.3%)	0.584
Beta-blockers (N, %)	6 (37.5%)	15 (68.2%)	0.099
Aldosterone antagonist (N, %)	11 (68.8%)	17 (77.3%)	0.713
Digoxin (N, %)	3 (18.8%)	10 (45.5%)	0.165
**Senescent T cell subsets**			
CD4^+^CD28^null^ T cell fraction in CD4^+^ T cell population (%)	4.5 ± 4.6	1.5 ± 1.7	0.027^*^
CD4^+^CD57^+^ T cell fraction in CD4^+^ T cell population (%)	6.9 ± 4.9	3.5 ± 1.9	0.019^*^
CD8^+^CD28^null^ T cell fraction in CD8^+^ T cell population (%)	43.2 ± 18.8	34.5 ± 15.9	0.134
CD8^+^CD57^+^ T cell fraction in CD8^+^ T cell population (%)	38.7 ± 16.1	31.9 ± 13.1	0.165

Values represent the number (%) or the mean ± standard deviation, as indicated. ^*^p-values < 0.05 are considered significant. CV, cardiovascular; BMI, body mass index; LVEF, left ventricular ejection fraction; LVEDD, left ventricular end-diastolic dimension; LVESD, left ventricular end-systolic dimension; LAVI, left atrial LAVI, left atrial volume index volume index; WBC, white blood cell; eGFR, estimated glomerular filtration rate; NT-proBNP, N-terminal of the prohormone brain natriuretic peptide; CRP, C-reactive protein; IL-6, interleukin-6; TNF-α, tumour necrosis factor-α; MIG, monokine-induced by gamma interferon; IP-10, interferon gamma-induced protein 10; MIP-1α, macrophage inflammatory protein-1α; RAS, renin angiotensin aldosterone system. Discrete variables were compared with the chi-squared method; continuous variables were compared with independent Student’s t-tests.

Finally, we examined the frequency of CD4^+^CD57^+^ T cells at the 6-month follow-up in 16 patients with available PBMCs. We found that patients with CV events showed significant elevations in CD4^+^CD57^+^ T cell frequencies at the 6-month follow-up compared to baseline frequencies (17.10 ± 14.75% vs. 8.70 ± 6.14%, *p* = 0.044). In contrast, patients without CV event showed no significant change in CD4^+^CD57^+^ T cell frequency over time (4.31 ± 3.01% vs. 3.30 ± 1.35%, *p* = 0.158; Fig. 4c). Regarding the association of gender and diabetic status with CD4^+^CD57^+^ T cell frequencies at baseline and at the six-month follow-up, there was no significant differences between male and female patients and between diabetic and non-diabetic patients, as shown in Supplementary Fig. 1.

## Discussion

Pathologic roles of CD4^+^ T cells in the development of HF have been well documented in recent animal studies^6–8^. In murine models of non-ischaemic, pressure-overload HF, activated CD4^+^ T cells were shown to infiltrate the myocardium and play a crucial role in promoting cardiac fibrosis, hypertrophy, and remodelling through cytokine release^6,7^. In a chronic ischaemic HF mouse model, the CD4^+^ T cell population was also expanded and activated. Moreover, studies in ischaemic HF models have shown that cardiac and splenic T cells were primed to induce cardiac injury and remodelling, and they retained this capability after an adoptive transfer^8^. Recently, Ramos *et al*. reported that the myocardial and immunological aging processes were intertwined phenomena, and that CD4^+^ T cells mediated cardiac inflammation and functional impairment in aged mice, even in the absence of clear tissue damage or concomitant infection^17^.

Previously, it was unclear which subsets of CD4^+^ T cells contributed to HF progression. In the present study, we demonstrated that CD4^+^CD57^+^ senescent T cells were elevated in patients with acute HF, and this elevation was associated with clinical outcome. Moreover, patients with acute HF had elevated levels of pro-inflammatory mediators, such as CRP, IL-6 and IP-10. A functional analysis of T cells from patients with acute HF revealed that the CD4^+^CD57^+^ T cell population exhibited a higher frequency of IFN-γ- and TNF-α- producing cells compared to the CD4^+^CD57^−^ T cell population. Furthermore, the frequency of CD4^+^CD57^+^ T cells at baseline and its elevation at the 6-month follow-up were significantly related to clinical outcome in patients with acute HF. All patients were treated with guideline-directed medical therapy during the follow-up period. Increased frequencies of CD4^+^CD57^+^ T cells in patients with a CV event may be caused by HF progression, considering the unchanged frequencies of CD4^+^CD57^+^ T cells in patients without CV events.

The clinical relevance of senescent CD4^+^ T cells in patients with chronic HF has been reported previously. Senescent CD4^+^ T cells were associated with chronic HF severity, and their frequency was an independent predictor of mortality^18^. In addition, senescent T cells were elevated in patients with an advanced clinical status of chronic HF, which suggested that they might contribute to disease progression^19^.

‘Immune aging’ or ‘immunosenescence’ can be briefly described by restriction of diversity, hypo-responsiveness to proper antigens, and enhanced non-specific pro-inflammatory responses^20^. A growing body of evidence has suggested that, in addition to HF, senescent T cells are involved in the pathogenesis of several cardiovascular diseases, including acute coronary syndrome^21–23^, myocardial infarction^13,24^, and hypertension^12,25,26^. Recently, we reported that CD8^+^CD57^+^ T-cell frequency was significantly higher in patients with pre-diabetes and type 2 diabetes compared to subjects with normal glucose tolerance, though CD4^+^CD57^+^ T-cell frequency was not^27^. In addition, we showed that the frequency of CD8^+^CD57^+^ T cells is an independent predictor of hyperglycaemia development^27^. The role of senescent T cells in the development hyperglycaemia and the progression of heart failure may be linked and partly explain the poor clinical outcome of diabetic HF patients.

Although inflammation is a major component of HF, clinical trials have shown that blocking TNF-α was not successful in controlling HF^4,5^. This failure might be attributable to the contributions of multiple mediators in the pathogenesis and progression of HF. Consequently, blocking the action of a single cytokine (e.g., TNF-α) might not be enough to control all the pathological immune responses involved in HF. Therefore, to identify more suitable targets for HF treatments, we must better characterize the immunological mechanisms involved in HF pathophysiology^28^. Currently, investigations in HF animal models are testing anti-inflammatory therapeutics with novel targets, including chemokine (C–C motif) receptor 2^29^, intercellular adhesion molecule 1^30^, and T-cell co-stimulators^31^. The results of the present study suggested that detailed studies on CD4^+^CD57^+^ senescent T cells in the context of HF might promote the development of novel anti-inflammatory therapeutics for HF treatment^32^.

The present study had several limitations. First, the study design was observational, rather than interventional. Thus, identifying a direct cause-and-effect relationship would be difficult. Relatively high baseline levels of pro-inflammatory mediators in control subject is another limitation of the observational study. Even though we enrolled age- and sex-matched control subjects who had no history of HF, the mean age of the control group was sixty-two and some of them had co-morbidities of hypertension and dyslipidemia which were well controlled. Old age and comorbidities might contribute to the increased level of pro-inflammatory mediators in these control subjects. Second, results from our study population are not generalizable to patients with compensated stable HF or patients with chronic HF that experience an acute exacerbation. Rather, our results pertained to treatment-naive patients with newly diagnosed acute HF. Third, T-cell changes at the 6-month follow-up could only be analysed in a small population, due to the limited availability of PBMCs. Fourth, the difference in clinical severity of HF may have affected the baseline leve of CD4^+^CD57^+^ T cells. However, we enrolled acute HF patients who recovered from the initial decompensation and were ready for discharge. Very sick patients who died in the index admission period were excluded from this study. Moreover, we sampled peripheral blood to measure mediators of inflammation and T-cell immunological characterization after patients achieved clinical stabilization according to current medical guidelines. Clinical stabilization was defined as readiness for discharge from the hospital: congestion was absent, and a stable, oral diuretic regimen established for at least 48 h. We collected blood samples at a relatively consistent time point, on the day of or before discharge. Finally, the small study population and the limited number of primary endpoints prevented a detailed and thorough investigation of whether the frequency of CD4^+^CD57^+^ T cells might serve as a prognostic factor in patients with acute HF. However, despite these limitations, our findings remain important, because we provided the first evidence to support a possible role of CD4^+^CD57^+^ senescent T cells in patients with acute HF.

## Supplementary information

Supplementary Figure

## Data Availability

The datasets used and/or analyzed during the current study available from the corresponding author on reasonable request.
