# Supplementary material for: Increased frequency of CD4+CD57+ senescent T cells in patients with newly diagnosed acute heart failure: exploring new pathogenic mechanisms with clinical relevance
Source: Sci Rep. 2019 Sep 9;9:12887. doi: 10.1038/s41598-019-49332-5 (PMC6733929; doi:10.1038/s41598-019-49332-5)
Supplement: Supplementary file 1 — Supplementary Figure [file 41598_2019_49332_MOESM1_ESM.docx]

Supplementary information

**Increased frequency of CD4^+^CD57^+^ senescent T cells in patients with newly diagnosed acute heart failure: exploring new pathogenic mechanisms with clinical relevance**

Jong-Chan Youn^1,2,*,⁑^, Min Kyung Jung^2,*^, Hee Tae Yu^2,3^, Ji-Soo Kwon^4^, Jeong-Eun Kwak^4^, Su-Hyung Park^4,5^, In-Cheol Kim^6^, Myung-Soo Park^7^, Sun Ki Lee^7^, Suk-Won Choi^7^, Seongwoo Han^7^, Kyu-Hyung Ryu^7^, Seok-Min Kang^3,⁑^, Eui-Cheol Shin^2,4,⁑^

**
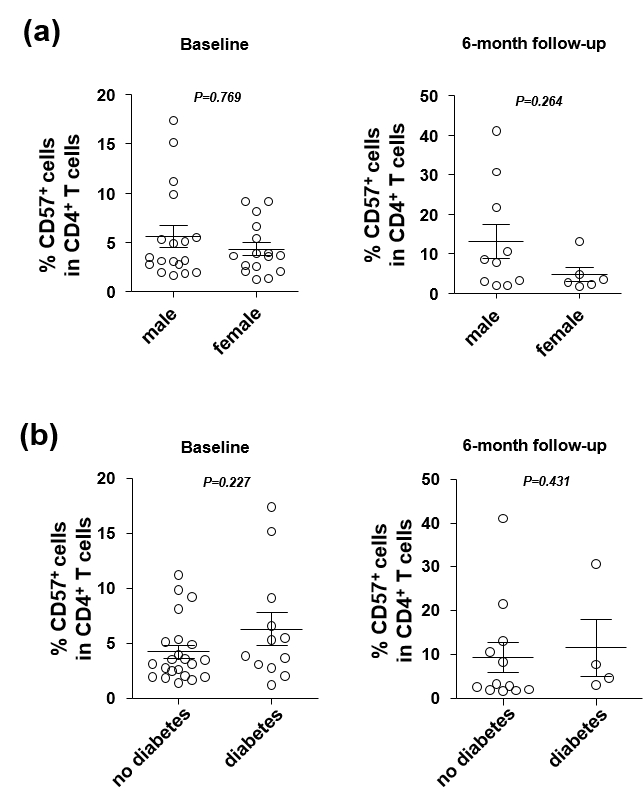
**

**Supplementary Figure S1. The frequencies of CD4^+^CD57^+^ T cells at baseline and at the 6-month follow-up according to gender and diabetic status showed no significant differences.**

(A) There was no significant differences for the frequencies of CD4^+^CD57^+^ T cells between male and female patients at baseline (p=0.769) and at the 6 month follow-up (p=0.264). (B) There was no significant differences for the frequencies of CD4^+^CD57^+^ T cells between diabetic and non-diabetic patients at baseline (p=0.227) and at the 6 month follow-up (p=0.431)
